# Switch controllers of an *n*-link revolute manipulator with a prismatic end-effector for landmark navigation

**DOI:** 10.7717/peerj-cs.885

**Published:** 2022-02-11

**Authors:** Ravinesh Chand, Ronal Pranil Chand, Sandeep Ameet Kumar

**Affiliations:** 1 School of Mathematical and Computing Sciences, Fiji National University, Suva, Fiji; 2 School of Information Technology, Engineering, Mathematics and Physics, University of the South Pacific, Suva, Fiji

**Keywords:** *n*-Link robotic arm, Switched system, Multiple Lyapunov functions, Velocity controllers, Hierarchal landmark

## Abstract

Robotic arms play an indispensable role in multiple sectors such as manufacturing, transportation and healthcare to improve human livelihoods and make possible their endeavors and innovations, which further enhance the quality of our lives. This paper considers such a robotic arm comprised of *n* revolute links and a prismatic end-effector, where the articulated arm is anchored in a restricted workspace. A new set of stabilizing switched velocity-based continuous controllers was derived using the Lyapunov-based Control Scheme (LbCS) from the category of classical approaches where switching of these nonlinear controllers is invoked by a new rule. The switched controllers enable the end-effector of the robotic arm to navigate autonomously *via* a series of landmarks, known as hierarchal landmarks, and finally converge to its equilibrium state. The interaction of the inherent attributes of LbCS that are the safeness, shortness and smoothness of paths for motion planning bring about cost and time efficiency of the controllers. The stability of the switched system was proven using Branicky’s stability criteria for switched systems based on multiple Lyapunov functions and was numerically validated using the RK4 method (Runge–Kutta method). Finally, computer simulation results are presented to show the effectiveness of the continuous time-invariant velocity-based controllers.

## Introduction

Human endeavors to handle tasks in harsh environments have brought many risks to the lives involved. The ever-increasing advancements in manufacturing and the demands of industrialization have given rise to specific robots designed to perform dangerous, dirty, and dull tasks in situations and environments which may be inaccessible or hazardous to humans (Sharma, Vanualailai & Singh, 2015). Industries worldwide adopt robots in applications such as sea rescue, and for supplementing retail and health care needs (Kumar, Vanualailai & Prasad, 2021; Kumar & Vanualailai, 2017; Lu et al., 2012; Gilmour et al., 2018). In scenarios where a human worker would struggle to perform safely, robots emerge to be an ideal candidate to accomplish tasks repetitively and consistently. The underlying intention of robotics is to build robots that can assist humans. It ensures jobs to be performed with a very high degree of accuracy and safety, and boasts the ability to deliver enhanced levels of service, reshaping lives and work practices. As such, robots have an invaluable role to play in the 21st century and beyond.

In the past few decades, there has been a serious approach to the study of robots and advancement by patrons, industries, academics, and researchers. This has been supported and facilitated by technological advances. As a result, a number of robotic mechanical systems such as aerial and ground vehicles, swimming and flying robots, parallel robots, car-like, tractor-trailer, and mobile manipulators have been researched. These studies have centred around different sectors in various real-world applications such as transportation (Gehrke, 2020; Raj et al., 2016; Raj et al., 2018; Chand et al., 2020), companionship (Mehta, Alim & Kumar, 2017), medical treatment and surgery (Gilmour et al., 2018), search and rescue (Kumar & Vanualailai, 2017), pursuit-evasion (Lin, Bekey & Abney, 2008), and explorations (Kumar et al., 2021; Kumar, Vanualailai & Prasad, 2020) to mention some.

Specific robots are designed to perform particular tasks, as different working environment in which robots operate in means that additional capabilities are required for optimum performance. In Mehta, Alim & Kumar (2017), it was shown that service robots such as wheelchairs are deployed as assistive technology in the healthcare system. Mechanical systems such as Segways used as personal transportation serve their purpose very well in constrained environments (Gehrke, 2020). Segways have been successfully used in courier and food delivery services. Such delivery robots can be used for both personal and professional services and bring convenience to home living (Jiao et al., 2020). In addition, the introduction and adoption of Uber services in American cities a decade ago has experienced substantial growth in its demand, as people take advantage by conveniently scheduling door-to-door, on-demand vehicle services offered by such app-based mobility services (Gehrke, 2020).

One valuable and prominent robotic system in the industrial sector is the robotic arm, which contributes to an improved production capacity of manufacturing companies (Lu et al., 2012). A robotic arm manipulator is an electronically controlled mechanism comprising multiple segments that perform tasks by interacting with its surroundings. The mechanical arm is usually programmable, with functions indistinguishable from a human arm (Chand, Kumar & Chand, 2021). It comprises of rigid links connected by several joints that either move along an axis or rotate in specific directions. Hence, classification of robotic arms is normally on the type of the links of the arm. The two commonly used joints are classified as prismatic and revolute. A prismatic joint provides a linear mechanism by allowing relative translation about an axis, whereas a revolute joint allows a relative rotation between two links (Aghajari, Dehkordi & Korayem, 2021). Different applications require robotic arms with different combinations of these joints.

The robotic arm has contributed significantly to many modern applications. A recent multi-factor application that has become quite popular in many developed countries is the pick and place robot. This manipulator can handle repetitive tasks with increased work rates, such as pickup and delivery in hospitals (Kumar, Sharma & Wu, 2018). In the manufacturing sector, assembly line robots have been programmed to put parts together, insert screws or dispense adhesives (Levitin, Rubinovitz & Shnits, 2006). Moreover, it has been shown in Bai et al. (2018) that robotic arms play a valuable role in search and rescue missions in areas of disaster, accidents, explosions, and terror attacks. In view of robot mobility, robotic arm types can also be categorized as anchored and non-anchored arm. An anchored robotic arm is a manipulator that performs its designated task from a fixed position, for example, industrial robots, which comprise a robot manipulator, power supply, and controllers as shown in Sacks (2003). In comparison, non-anchored robotic arms are an active component of mobile manipulators (Prasad et al., 2020a), contributing to several real-life applications such as mining, forestry, planetary exploration, and the military (Lin, Bekey & Abney, 2008; Kumar et al., 2021; Grehl, Mischo & Jung, 2017; Parker, Bayne & Clinton, 2016).

The techniques, strategies, and methods utilized to solve such problems are varied. These methods are commonly categorized under cell decomposition (Rosell & Iniguez, 2005), visible graph (Lozano-Pérez & Wesley, 1979), artificial potential field (Cosío & Castaneda, 2004), probabilistic roadmap (Wahab, Nefti-Meziani & Atyabi, 2020), and Dubins path (Dubins, 1957). In the cell decomposition method, the free space of the robot’s configuration is divided into smaller regions called cells. The goal is to provide a collision-free path to reach the target. The applications of robot path planning based on this approach can be found in Rosell & Iniguez (2005). If a free path exists, exact cell decomposition will find it; however, the trade-off for this accuracy is a more difficult mathematical process. The visible graph is a traditional method of path planning to find Euclidean shortest paths among a set of polygonal obstacles in the plane. It is a graph of intervisible locations, typically for a set of points and obstacles in the Euclidean plane, according to Lozano-Pérez & Wesley (1979). The weakness of this method is that if the environment contains many obstacles, the computing time is increased, while the resultant part of the path planning is also not safe. In the artificial potential field method, the obstacles and the goals are assigned repulsive and attractive forces, respectively, so the robot can move towards its target while avoiding obstacles (Cosío & Castaneda, 2004). The major draw back is that algorithm singularities (local minima) are introduced. However, the method has been commonly deployed in research and design as it is simple to use and easy to implement (Cosío & Castaneda, 2004).

Although the robotic arm works well for single tasks, such as pick and place; however, a series of tasks to be performed demands the availability of a high volume of information. This implies that a set of information packets need to be processed one after another as the robot continues to operate in the workspace. The information can be translated into visual or sensory cues, which robots can use to reach a target as in pick and place robots (Kumar, Sharma & Wu, 2018) or to perform intermediary tasks as in assembly line systems (Levitin, Rubinovitz & Shnits, 2006).

Motion planning and control of robots is vital to ensure successful accomplishment of designated tasks. Numerous bio-inspired behaviors and features have been borrowed from nature and incorporated into robotics for improved motion planning and control of robots (Gravish & Lauder, 2018). Yet another noteworthy feature is a landmark, an assisting feature to aid motion or movement. The landmark technique is naturally employed by insects and animals (Prasad et al., 2020b) and can be incorporated into algorithms or control laws of manipulators to navigate a goal position in known or unknown environments. The landmark technique has been used in studies such as Prasad, Sharma & Kumar (2020) and Prasad et al. (2020b) to guide a robot to the desired goal and for self-localization, where subgoals for a planning problem are landmarks. As described by Prasad et al. (2020b), landmarks are mandatory tasks that any defined solution plan should perform. One way to attain the maximum benefit from the landmark technique is by performing in a hierarchy; task 1 must be completed before performing task 2 (Prasad, Sharma & Kumar, 2020). For instance, the task 2 of a delivery robot is to deliver a food pack after picking it, making picking the food pack task 1. As a result, landmarks can be sanctioned according to the order they need to be executed. This technique, where the landmarks are provided to the robot in a hierarchal order, is known as hierarchal landmarks, and has been effectively utilized in Prasad et al. (2020b) where landmarks are ordered, and the first landmark is to be reached before moving to the second landmark, and so on.

This research is motivated by the gap seen in the literature on the presence of an *n*-link robotic arm to address real-life problems under the guidance of landmarks. In this paper, the stabilizing velocity controllers of an *n*-link revolute robotic arm with a prismatic end-effector $({R^n}P$) are developed. The end-effector navigates *via* hierarchal landmarks, which act as waypoints, to perform multiple assigned tasks. Consequently, navigating to a unique hierarchal landmark creates a separate subsystem, and a combination of such distinct subsystems develops a switched system. Switched systems are referred to as hybrid dynamical systems that consist of a group of continuous-time subsystems and typically include a specific rule that facilitates the switching (Liberzon & Morse, 1999). Therefore, the stabilizing switched velocity controllers will ensure that the end-effector of the ${R^n}P$ robotic arm successfully maneuvers from its initial configuration in a *priori* known environment *via* different hierarchal landmarks to its final configuration. This innovative method can have real-life health care, assembly-line production, logistics, and military applications. For instance, in the manufacturing sector, industrial robots used for the assembly of parts use fixed or moving landmarks for point recognition in the workspace to navigate safely to its target. The velocity based-controllers for the ${R^n}P$ robotic arm are derived for each subsystem using the method of Lyapunov-based Control Scheme (LbCS).

The main contributions of this paper are:

1. Navigation of an ${R^n}P$ robotic arm through hierarchal landmarks. This technique enables the robot arm to perform a sequence of tasks, navigating amongst the hierarchal landmarks. In contrast, the robotic arms reported in the literature perform single task in the workspace (Chen et al., 2019; Kim, Choi & Kim, 2013).
2. A new dynamic and generalized *n*-link revolute robotic arm with a prismatic end-effector. However, the literature survey reveals that a specific number of links were utilized for revolute robotic arms, for instance, 2-link, 3-link, 5-link, and 6-link mechanisms were studied in Meyer (1993), Sharma, Vanualailai & Prasad (2011), Martinetz, Ritter & Schulten (1990), Iqbal, Islam & Khan (2012) and Asadi et al. (2019) respectively.
3. Time-invariant, stabilizing, switched nonlinear, and continuous velocity controllers of ${R^n}P$ robotic arm. From the authors’ point of view, this is the first time such stabilizing velocity-based controllers are derived for ${R^n}P$ robotic arm in the sense of Lyapunov.
4. A robust system due to inherent nature of the LbCS control scheme which enables the system to respond well to the singularities and limitations of the mechanical system. The singularities and limitations are treated as artificial obstacles in the control scheme. Unlike the robotic arms, such as ones reported in Cosío & Castaneda (2004), Guez & Ahmad (1988) and Jack et al. (1993), where irregularities in the singularities resulted in unstable motion and velocities, subsequently leading to the systems’ failure.

In “Literature Review”, a discussion on the literature review is presented. “Lyapunov-based Control Scheme” gives a brief description of the Lyapunov-based Control Scheme, while in “System Modeling”, the system modeling of an *n*-link revolute robotic arm with a prismatic end-effector is shown. “Findpath Problem Via Hierarchal Landmarks” discusses the find-path problem *via* landmarks for an *n*-link robot arm. In “Lyapunov-based Velocity Controllers”, the switched velocity controllers are derived from multiple Lyapunov functions and the stability analysis of the *n*-link robot arm is discussed. Then, in “Simulation Results”, the simulation results are presented, followed by discussion and conclusion in “Discussion” and “Conclusion”, respectively.

## Literature Review

In the recent past, several notable developments have been made in the field of robotic arms. As a result of such developments, robotic arms have continuously and successfully contributed to the growth of the medical and industrial sectors. Researchers have employed various techniques to control the motion of anchored and unanchored manipulators, subsequently increasing the number of links in the system for improved performances.

Casting a quick look some three decades back discloses that researchers initially focussed on 2-link and 3-link manipulators, utilizing numerous methods for motion control of robotic arms. In 1993, Meyer first worked on path planning using the unanchored robotic arm, where he studied the find-path problem using algorithms based on the velocities of different components of the 2-link planar revolute arm (Meyer, 1993). In 2003, Sacks (Sacks, 2003) used configuration spaces and compliant motion to study route planning for planar articulated industrial robots. In 2011, Sharma, Vanualailai & Prasad (2011) used Lyapunov-based decentralized formation control planner for a swarm of 2-link mobile manipulators. To ensure stability, nonlinear control laws were extracted and utilized from LbCS to obtain collision-free trajectories of the swarm. In Martinetz, Ritter & Schulten (1990) used Kohonen networks to develop a learning algorithm for the visuomotor coordination of a 3-link revolute arm, in Guez & Ahmad (1988) used neural networks on a 3-link manipulator to test mappings from both cartesian and spherical coordinates to manipulator joint coordinates, and in Josin, Charney & White (1988) used neural networks and inverse kinematics for motion planning on a 2-link planar manipulator that is mainly restricted to small areas in the center of a workspace. Moreover, inverse kinematics algorithm was utilized by Megalingam et al. (2017) to prescribe the motion of the 3-link robotic arm having prismatic joints. In Jack et al. (1993) demonstrated that for a 3-link, 3 degrees of freedom (3-DOF) manipulator, the inverse kinematics function can be approximately represented using an artificial neural network. The integration of device singularities into the proposed solution was a significant weakness in Guez & Ahmad (1988) and Jack et al. (1993), as irregularities in the singularities mainly resulted in the systems’ failure.

To attain stable and controlled motion of robotic arm systems, the method of LbCS was also utilized. Vanualailai, Nakagiri & Ha (1998) used the Lagrange method with a set of differential equations administering the planar robot system and proposed a solution for the motion control using LbCS. In 2014, Prasad, Sharma & Vanualailai (2014) derived velocity-based controllers to solve the find-path problem of a three dimensional 2-link revolute articulated manipulator arm. Prasad et al. (2021) showed how to solve the motion control of a 3-dimensional articulated mobile manipulator in the presence of fixed obstacles using the Direct Method of Lyapunov.

Research in this area gained momentum in 2012 as additional links were introduced to the robotic arm systems, resulting in the publication of a number of articles. Iqbal, Islam & Khan (2012) kinematically modeled and analyzed the workspace of the widely used 5-link, 6-DOF revolute robotic arm manipulator, ED7220C. However, in his study, position precision could not be achieved accurately due to inappropriate joint angles resulting from the improper mechanical coupling of the joints and non-linearity in mapping angles. To achieve the stability of the robotic system, Sharma, Vanualailai & Singh (2012, 2015*)* presented decentralized continuous acceleration controls for motion planning and posture control of a single (Sharma, Vanualailai & Singh, 2012) and multiple (Sharma, Vanualailai & Singh, 2015) *n*-link doubly nonholonomic mobile manipulators avoiding obstacles. A robotic arm controller was presented in Jahnavi & Sivraj (2017) that allowed for the use of an anchored 5-link robot arm as a practical laboratory model for teaching and learning robot arm algorithms. The authors of Serrezuela et al. (2017) attempted to mathematically examine a 4-link manipulator robot arm’s performance and position with respect to the joint angles related to a coordinate system. Besides, Avanzini, Zanchettin & Rocco (2018) in 2017 developed a controller based on constrained optimization for tracking problems of non-anchored mobile manipulators.

Furthermore, Carron et al. (2019) presented a novel approach for controlling a 6-link revolute robotic arm based on a data-driven model predictive controller. Asadi et al. (2019) took advantage of technological advancements and proposed a mobile unmanned ground vehicle (UGV) equipped with a stereo camera and a 6-link revolute robotic arm that could remove obstacles along the UGV’s path. In the study, while the 6-DOF system was able to track the location and orientation of the detected objects, the arm failed to reach the object by following the predetermined orders.

A notable contribution of robotic arms is their application as pick and place robots. In Kim, Choi & Kim (2013), the authors conducted an independent flight experiment that comprehended picking up and delivering an object, which requisite accurate control of a quadrotor and 2-link revolute robot arm. The experimental results were not impressive, as they showed that the designed tasks could be accomplished only satisfactorily. In 2019, Chen et al. (2019) controlled a 3-link revolute robotic arm for performing pick and place tasks that require control with multiple degrees of freedom. However, to enhance its feasibility in practical situations, the proposed system needed more robust computer vision to identify objects in the workspace. All these findings revealed that further work needed to be done, more specifically to refine the robotic system’s algorithms in order to enhance its performance in regards to performing a sequence of tasks, and subsequently to use them in real-world applications.

Robotics and technology-enabled environments play a critical role in helping elderly and physically disabled people to remain self-sufficient and autonomous in their familiar surroundings. This motivated Bassily et al. (2014) to develop a human-machine communication interface between the Leap Motion controller and the 6-link Jaco robotic arm with revolute joints in 2014. Further work on this scope continued, and a couple of years later, Kruthika, Kumar & Lakshminarayanan (2016) presented the design of an unanchored 5-link revolute robotic arm with 5-DOF that was used to feed the elderly or specially challenged. The robotic arm was operated and controlled using robot kinematics principles and MATLAB. In 2018, significant research was carried out in the medical sector as seen in Gilmour et al. (2018), Kopperger et al. (2018), Kayani et al. (2018) and Kayani et al. (2018). These work particularly centralized in introducing robotic-arm assisted unicompartmental knee arthroplasty into routine surgical practice. The COVID-19 outbreak in 2020 has resulted in the manufacturing and service sectors being negatively affected across the globe. Different types of robots are deliberated upon in Javaid et al. (2020) on their use to productively deliver medicine, food, and other essential items to COVID-19 patients in certain quarantine facilities around the world.

This literature search reveals an unexplored concept based on an *n*-link robotic arm to address real-life problems under the guidance of landmarks. The manipulator proposed in this paper uses the method of LbCS and has the ability to navigate *via* hierarchal landmarks to its target. This novel technique can potentially have real-life applications in military, health care, logistics, and assembly line production.

## Lyapunov-Based Control Scheme

Sharma, Vanualailai & Prasad (2017) proposed the Lyapunov-based Control Scheme (LbCS), which falls under the artificial potential field method, widely used in robotics research for motion planning and control of robotic systems. The time-invariant nonlinear velocity or acceleration controllers can be derived from LbCS, which has been successfully applied in the literature to find feasible and stabilizing solutions to a variety of problems (Sharma, Vanualailai & Singh, 2015; Sharma, Vanualailai & Singh, 2014; Kumar, Vanualailai & Sharma, 2016; Chand, Kumar & Chand, 2021; Sharma et al., 2018; Kumar et al., 2021; Sharma, Raj & Vanualailai, 2018).

LbCS includes the design of attractive and obstacle avoidance functions for target attraction and repulsion from various obstacles, respectively. The repulsive potential functions are designed as ratios with the obstacle avoidance function in the denominator of each ratio and a positive tuning parameter in the numerator.

In contrast, the attractive functions can be treated as attractive potential field functions. The total potentials, also known as Lyapunov functions, comprises the sum of these potential functions, and used to design the velocity or acceleration controllers of the mechanical system under study. The method’s guiding principle is to apply an attractive field to the target and a repulsive field to each obstacle. The entire workspace is then inundated with positive and negative fields, with the concept of steepest descent facilitating motion direction.

The input force, or the gradient of total potential, defines the speed and direction in which the mechanical system moves. Designing controllers with LbCS is easy, and the controllers are continuous, which is one of the scheme’s key advantages. The main drawback of LbCS is the possibility of algorithm singularities (local minima). In real-life applications, continuity has to be discretized, and asymptotic stability can only be demonstrated. A detailed account of the LbCS could be found in Sharma, Vanualailai & Prasad (2017) and Sharma, Vanualailai & Singh (2014).

**Figure 1:**
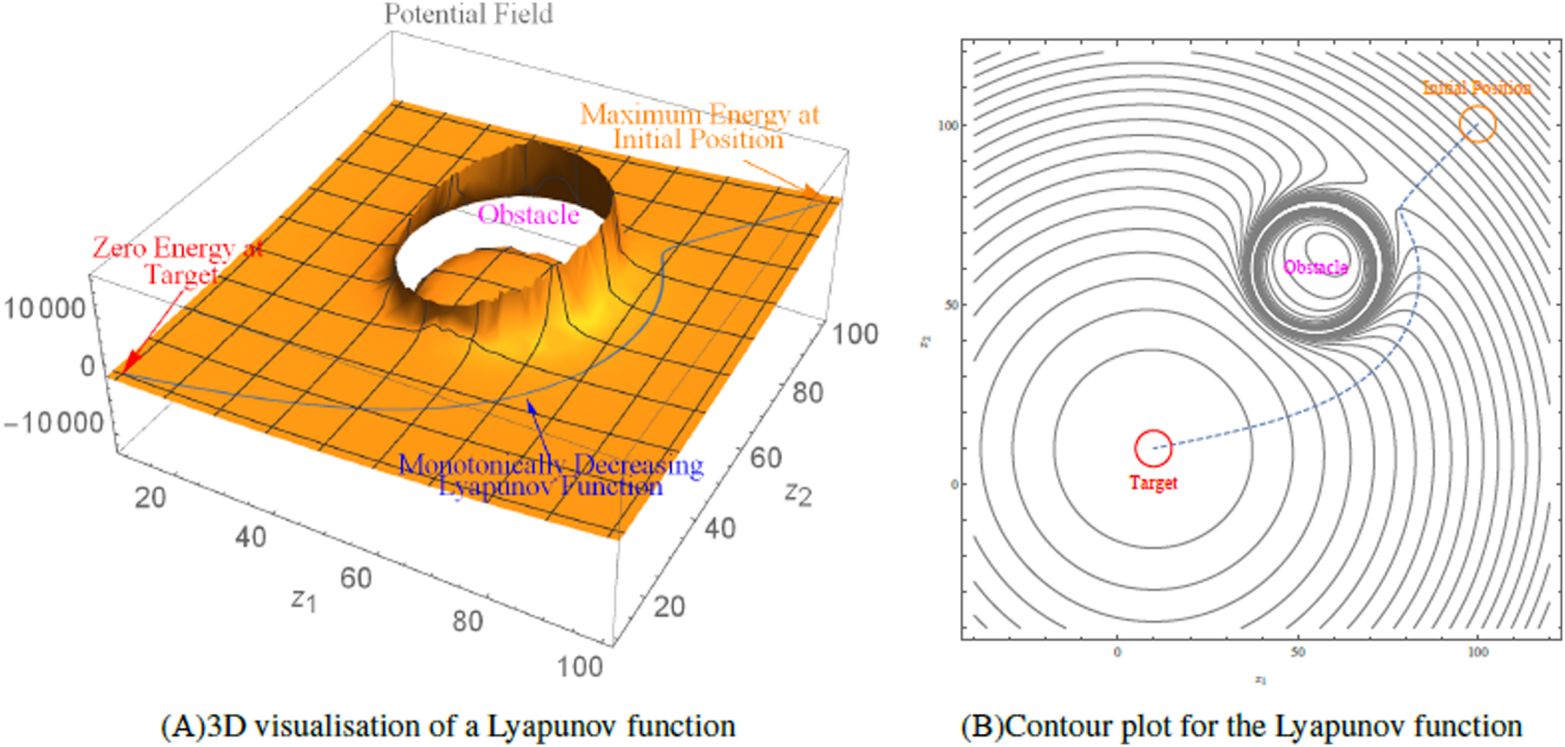
An illustration of the Lyapunov-based control scheme.

Figure 1 depicts the contour plot and 3D visualisation of a Lyapunov function created over workspace for a robot whose initial position is at (100,100). The robot’s trajectory from its initial location to its target position (10, 10) is depicted by the dotted line, which shows the robot avoiding the obstacle at (65, 60) with radius 20.

## System Modeling

**Figure 2:**
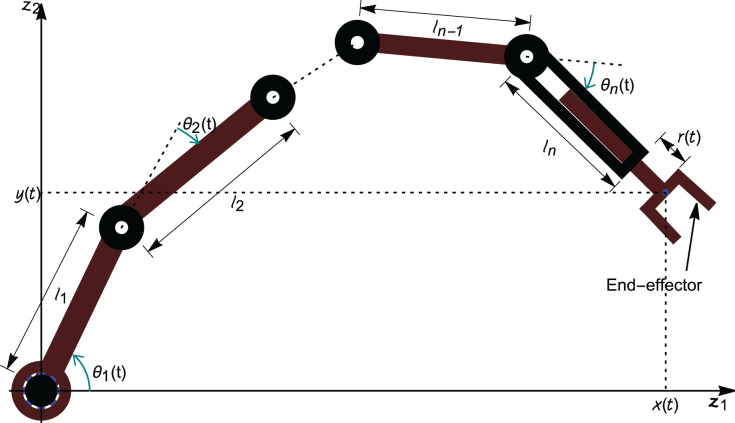
Schematic representation of an *n*-link revolute manipulator with a prismatic end-effector.

An *n*-link revolute manipulator arm is considered in the research, having *n* rotational joints in the ${z_1}{z_2}$-plane as shown in Fig. 2. The articulated arm consists of *n* rigid links which are connected *via* revolute joints and the *n*^th^ link has a prismatic joint with an end-effector.

With the help of Fig. 2, it is assumed that:

- the planar ${R^n}P$ manipulator is anchored at the origin;
- the length of the ${i^{th}}$ revolute link is ${l_i}$ with an angular position ${\theta _i}(t)$ at time $t$ with the horizontal ${z_1}$ axis;
- the last link (link $n$) has the length ${l_n} + r(t)$ with an angular position ${\theta _n}(t)$ at time $t$; and
- the coordinates of the gripper is $(x(t),y(t))$ and is given as:

$\matrix{ {x(t)}  & =  & {\sum\limits_{i = 1}^{n - 1} {{l_i}} \cos \left( {\sum\limits_{k = 1}^i {{\theta _k}} (t)} \right) + ({l_n} + r(t))\cos {\mkern 1mu} \left( {\sum\limits_{k = 1}^n {{\theta _k}} (t)} \right),}  \cr {y(t)}  & =  & {\sum\limits_{i = 1}^{n - 1} {{l_i}} \sin \left( {\sum\limits_{k = 1}^i {{\theta _k}} (t)} \right) + ({l_n} + r(t))\sin {\mkern 1mu} \left( {\sum\limits_{k = 1}^n {{\theta _k}} (t)} \right).}  \cr }$

Next, a system of differential equations is constructed that describe the motion of *n*-link revolute manipulator arm with a prismatic end-effector. Let the position of the end-effector of the *n*-link robot arm at $t \ge 0$ be ${\bf x} = (x(t),y(t))$ with an orientation angle of ${\theta _n} = {\theta _n}(t)$. Let the angular orientation of the ${i^{th}}$ link be ${\theta _i} = {\theta _i}(t)$ for $i \in \{ 1,2,3...,n - 1\} .$ Also,

$$\matrix{ x(t) = {\rm the }\, x - \text{component of the position of the end-effector} \cr y(t) = {\rm the }\, y - \text{component of the position of the end-effector} \cr {\theta _i}(t) = \text{the angular position of the}\, \rm{i}^{th}\, {\rm link\, for }\, i = 1,2,3,\ldots ,n \cr {w_i}(t) = \text{the angular velocity of the}\, \rm{i}^{th}\, {\rm link\, for }\, i = 1,2,3,\ldots ,n \cr v(t) = \text{the linear velocity of the}\, \text{prismatic}\, {\rm link} }$$

$\forall t \ge 0$. Thus, the kinematic model of the *n*-link robot

arm (on suppressing *t*) is as follows:

(1) $$\left. {\matrix{ {\dot x = v\cos \left( {\sum\limits_{k = 1}^n {{\theta _k}} } \right) - \sum\limits_{i = 1}^n {{w_i}} y + \sum\limits_{i = 2}^n {{w_i}} \sum\limits_{j = 1}^{i - 1} {{l_j}} \sin {\mkern 1mu} \left( {\sum\limits_{k = 1}^j {{\theta _k}} } \right),} \cr {\dot y = v\sin \left( {\sum\limits_{k = 1}^n {{\theta _k}} } \right) + \sum\limits_{i = 1}^n {{w_i}} x - \sum\limits_{i = 2}^n {{w_i}} \sum\limits_{j = 1}^{i - 1} {{l_j}} \cos {\mkern 1mu} \left( {\sum\limits_{k = 1}^j {{\theta _k}} } \right),} \cr {{{\dot \theta }_i} = {w_i},} \cr {\dot r = v.} \cr } } \right\}$$

At $t \ge 0$, let $({w_i}(t),v(t)): = ({{\theta }^{\prime}_i}(t),{r}^{\prime}(t))$ be the instantaneous angular velocity of the ${i^{th}}$ revolute link and linear velocity of the prismatic end-effector. Thus, a system of first-order ordinary differential equations (ODEs) for the ${i^{th}}$ revolute link and the prismatic end-effector is obtained as:

(2) $${{\theta }^{\prime}_i}(t) = {w_i}(t),\;{r}^{\prime}(t) = v(t),$$

assuming the initial conditions at $t = {t_0} \ge 0$ as ${x_0}: = x({t_0}),\;{y_0}: = y({t_0}),\;{\theta _{i0}}: = {\theta _i}({t_0}),\;{r_0} = r({t_0}).$ Let ${{\bf x}_0} = ({x_0},{y_0})$.

Suppressing $t$, the state vector is given as ${\bf q}: = (x,y,{\theta _1},{\theta _2}, \ldots ,{\theta _n},r) \in {{\mathbb R}^{n + 3}}$. Also let ${{\bf q}_0}: = {\bf q}({t_0}): = ({x_0},{y_0},{\theta _{{1_0}}},{\theta _{{2_0}}}, \ldots ,{\theta _{{n_0}}},{r_0}) \in {{\mathbb R}^{n + 3}}$. If the state feedback law of the instantaneous velocity $({w_i},v)$ has the form

$\eqalign{& {w_i}(t): = - {\mu _i}{f_i}({\bf q}(t)), \cr & v(t): = - \varphi g({\bf q}(t)),}$

for $i \in \{ 1,2,3, \ldots ,n\}$, for some scalars ${\mu _i},\varphi$ and some functions ${f_i}({\bf q}(t))$, and $g({\bf q}(t))$, to be constructed appropriately later, and if we define ${\bf F}({\bf q}): = ( - {\mu _1}{f_1}({\bf q}), - {\mu _2}{f_2}({\bf q}), \ldots , - {\mu _n}{f_n}({\bf q}), - \varphi g({\bf q})) \in {{\mathbb R}^{n + 1}}$, then the *n*-link manipulator with a prismatic end-effector is represented by

(3) $$\mathop {\bf q}\limits^. = {\bf F}({\bf q}),\;\;{\bf q}({t_0}) = {{\bf q}_0}.$$

## Findpath Problem Via Hierarchal Landmarks

**Figure 3:**
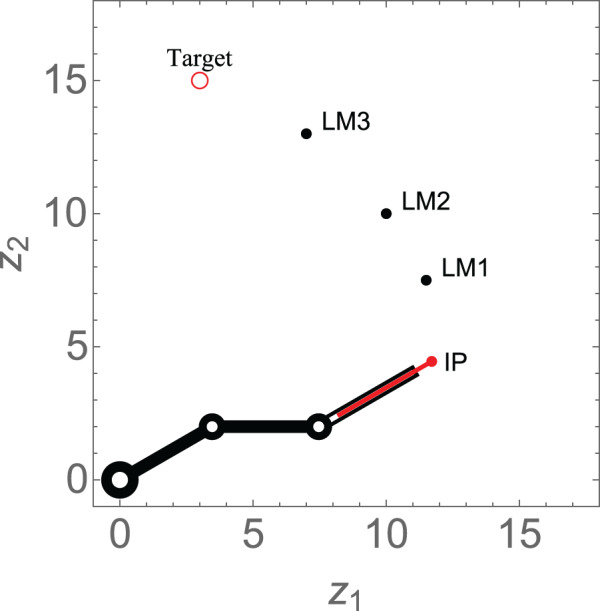
Schematic design of the findpath problem for a 3-link revolute manipulator with a prismatic end-effector *via* hierarchal landmarks.

Consider a workspace of an *n*-link revolute manipulator with a prismatic end-effector with predefined $m \in {\mathbb N}$ hierarchal landmarks. Assume that the locations of the $m \in {\mathbb N}$ hierarchal landmarks, $L{M_p}$ for $p \in \{ 1,2, \ldots ,m\}$, have already been determined. As illustrated in Fig. 3, the prismatic end-effector of a 3-link revolute manipulator has to navigate to the target location *via* the three hierarchal landmarks.

**Figure 4:**
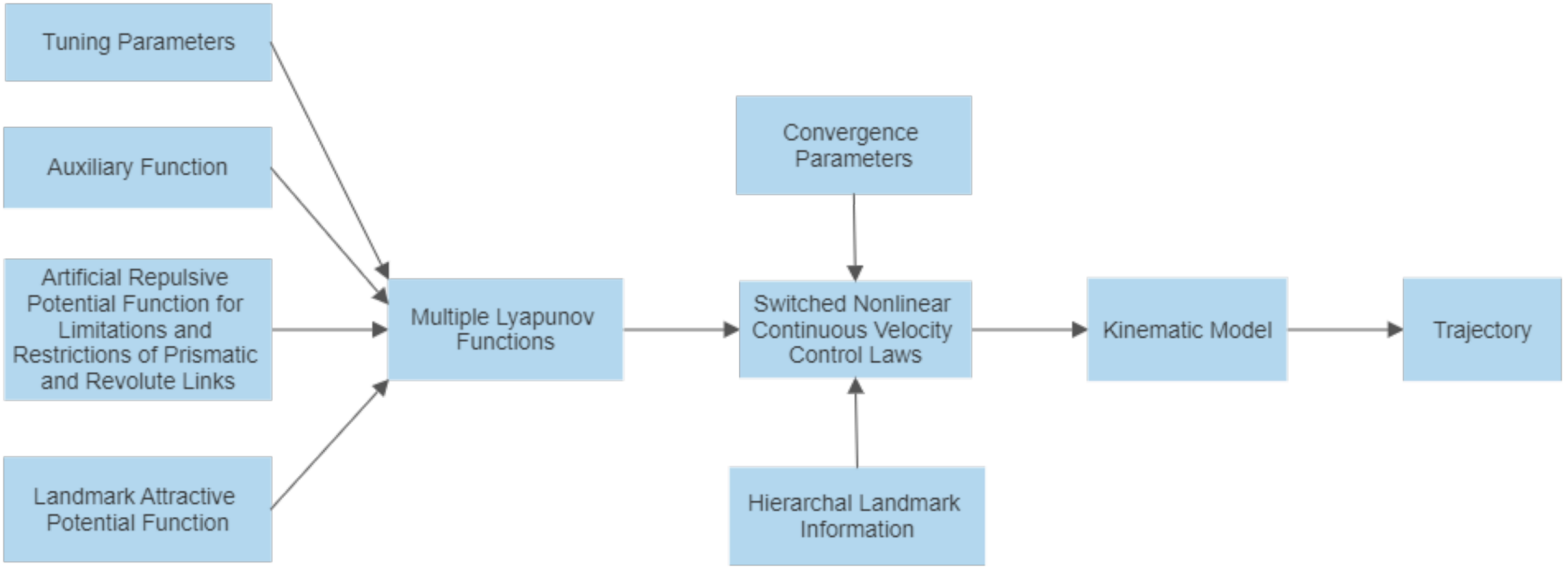
Block diagram illustrating the control scheme.

Before reaching its ultimate destination, the system must perform assigned tasks at each of these hierarchal landmarks. For example, if a pick and place robot needs to place an object at the first landmark, an object at the second landmark, another at the third landmark, and so on, until the ultimate target is reached, it implies that a sequence of tasks is being carried out. This technique will enable the robot arm to perform a sequence of tasks with one complete movement of the articulated arm. The design of the switched nonlinear continuous velocity control laws is captured in Fig. 4.

**Definition 5.1** *The* ${p^{th}}$ *landmark* $L{M_p}$, *for* $p = 1,2,...,m$, *is a disk with center* ${{\bf x}_{L{M_p}}} = ({x_{L{M_p}}},{y_{L{M_p}}})$ *and radius* ${r_{L{M_p}}}$ (Kumar et al., 2021). *The set* $L{M_p}$ *is defined as:*

(4) $$L{M_p} = \{ ({z_1},{z_2}) \in {{\mathbb R}^{\rm 2}}:{({{\rm z}_{\rm 1}}{\rm - }{x_{L{M_p}}})^2} + {({z_2} - {y_{L{M_p}}})^2} \le r_{L{M_p}}^2\} .$$

**Definition 5.2** *The final target for the end-effector of n-link robot arm is a disk with the center* ${{\bf x}_\tau } = (a,b)$ *and radius* ${r_\tau }$ (Kumar et al., 2021). *It is described as the set:*

$\tau = \{ ({z_1},{z_2}) \in {{\mathbb R}^{\rm 2}}:{({{\rm z}_{\rm 1}}{\rm - a})^{\rm 2}}{\rm + }{({{\rm z}_{\rm 2}}{\rm - b})^2} \le r_\tau ^2\} .$

The target ${{\bf x}_\tau }$ will be considered as an additional landmark $(L{M_{m + 1}} = {{\bf x}_\tau })$. The distance, ${\lambda _{L{M_p}}}$, between the initial position of the robot end-effector, ${{\bf x}_0}$, and the $p$th landmark, is given by:

(5) $${\lambda _{L{M_p}}} = \left\| {{{\bf x}_0} - {{\bf x}_{L{M_p}}}} \right\|,$$

*for* $p \in \{ 1,2,3,...,m + 1\}$.

It is further assumed that

${\lambda _{L{M_1}}} < {\lambda _{L{M_2}}} < {\lambda _{L{M_3}}} < ... < {\lambda _{L{M_{m + 1}}}}.$

**Definition 5.3** *The equilibrium point for the* ${n^{th}}$ *link is defined as:*

${{\bf q}_e}: = (a,b,{\theta _{{1_f}}},{\theta _{{2_f}}}, \ldots ,{\theta _{{n_f}}},{r_f}) \in {{\mathbb R}^{n + 3}},$

*where* ${\theta _{{i_f}}}$ *represents the final orientation angle of the* ${i^{th}}$ *revolute link for* $i \in \{ 1,2, \ldots ,n\}$ *and* ${r_f}$ *is the final extracted length of the prismatic end-effector*.

## Lyapunov-Based Velocity Controllers

The design of the controllers is generalised to cover *n*-links. The design, control algorithm and switch control remains the same for manipulators with different link numbers. The control design, as seen from the algorithms, will increase or decrease the number of inputs or outputs based on the number of links. However, the control design is generic enough to cover for any number of links.

### Components of multiple Lyapunov function

In the multiple Lyapunov functions to be proposed, the following potential functions will be included.

#### Landmark attraction function

The attractive potential that would ensure that the end-effector of the *n*-link robotic arm maneuvers to the ${p^{th}}$ hierarchal landmark, is proposed to be:

(6) $${V_p}({\bf x}) = \displaystyle{1 \over 2}{\left\| {{\bf x} - {{\bf x}_{L{M_p}}}} \right\|^2},$$

for $p \in \{ 1,2, \ldots ,m\}$.

#### Auxiliary function

To ensure that the end-effector of the robotic arm converges to its equilibrium position, radically unbounded functions about the target are utilized.

(7) $$H({\bf x}) = \displaystyle{1 \over 2}{\left\| {{\bf x} - {{\bf x}_\tau }} \right\|^2}.$$

#### Limitations and restrictions

**Figure 5:**
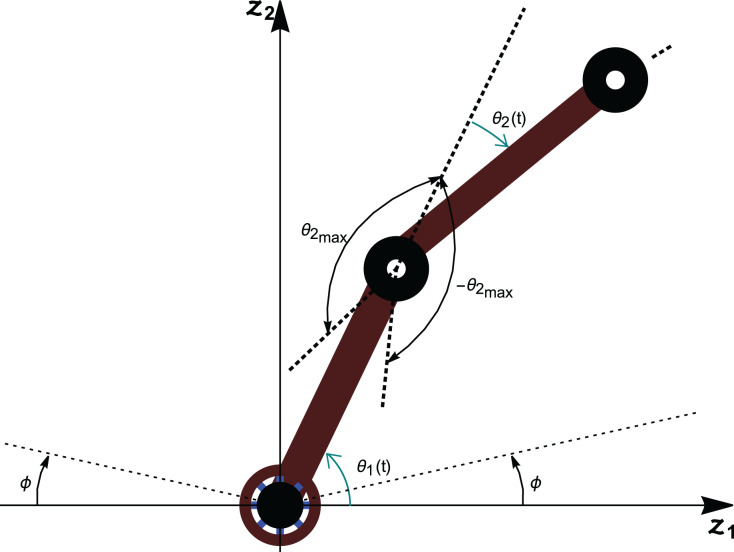
Limitations of the revolute links.

There is a need to account for all singularities created from the anchored arm’s geometric arrangement. Link 1 of the anchored arm cannot rotate fully to the horizontal surface on which it is mounted on when rotating both clockwise and counterclockwise. The singularities of link 1 arise when ${\theta _1} \in \{ \phi ,\pi - \phi \}$ as shown in Fig. 5.

### Assumption

Link 2 to link *n* can fully stretch and can rotate both clockwise and counter clockwise.

Link 2 cannot fully fold with link 1 while rotating both clockwise and counterclockwise. This singularity of link 2 arise when ${\theta _2} = \left| {{\theta _{{2_{max}}}}} \right|$ as shown in Fig. 5. The same singularity arises for other revolute links. Thus, it could be generalized as ${\theta _i} = \left| {{\theta _{{i_{max}}}}} \right|$ for $i \in \{ 2,3, \ldots ,n\}$. Moreover, the prismatic link cannot be fully extracted or withdrawn. The singularities of the prismatic link arise when $r(t) \in \{ 0,{r_{max}}\}$. These internal singularities occur in the workspace configurations that cannot reduce the holonomic constraints of the system, and will be avoided by considering them as artificial obstacles in accordance with LbCS.

i. **Angular Limitation and Restriction**

To avoid the singularities of the first link of the revolute arm, the following functions are introduced:

${W_1} = \phi - {\theta _1}{\mkern 1mu}\ {\rm and}\ {\mkern 1mu} {W_2} = \pi - \phi - \left| {{\theta _1}} \right|.$

To avoid interior singularities of the other revolute links, consider the function:

${W_{i + 1}} = {\theta _{{i_{max}}}} - \left| {{\theta _i}} \right|$

for $i = \{ 2,3,..,n\}$.

ii. **Prismatic link Length Limitation and Restriction**

The end-effector of the prismatic link can not go inside the last revolute link. The translational link restriction is that $r(t) \ne 0$ at any time *t*, that is ${r_0} = r(0) > 0$. The limitation is that ${r_{max}} - r(t) \ge {r_0}$ at any time *t*. To avoid this singularity, the following functions are defined:

$\matrix{ {{\eta_1}} = {r(t){\mkern 1mu} \; and\; {\mkern 1mu} {\eta _2}} = {{r_{max}} - r(t),}  \cr }$

where ${r_{max}}$ is the total length of the prismatic link.

### Multiple Lyapunov functions

Let $\alpha ,{\delta _p},{\beta _i}$ and ${\gamma _i}$ be positive real numbers, and let $\lambda = \left\| {{\bf x} - {{\bf x}_{L{M_p}}}} \right\|$. Define for $i \in \{ 1,2,3,...,n\}$ a group of Lyapunov functions of the form,

(8) $${L_p}({\bf q}) = H({\bf x})\left( {\alpha + {\delta _p}{V_p}({\bf x}) + \sum\limits_{i = 1}^2 {\displaystyle{{{\gamma _i}} \over {{\eta _i}}}} + \sum\limits_{i = 1}^{n + 1} {\displaystyle{{{\beta _{\rm i}}} \over {{W_i}}}} } \right),$$

which will operate according to the switching rule

(9) $p = \left\{ {\matrix{ 1 & {0 \le \lambda \lt {\lambda _{L{M_1}}}} \cr 2 & {{\lambda _{L{M_1}}} \le \lambda \lt {\lambda _{L{M_2}}}} \cr 3 & {{\lambda _{L{M_2}}} \le \lambda \lt {\lambda _{L{M_3}}}} \cr \vdots & {} \cr {m + 1} & {{\lambda _{L{M_m}}} \le \lambda \le {\lambda _{L{M_{m + 1}.}}}} \cr } }\right. $$$

### Velocity controllers

Along a trajectory of system (3),

(10) $$\eqalign{
  & {{\dot{L}}_p}({\bf{q}}) = \dot{H}({\bf{x}})\left( {\alpha  + {\delta _p}{V_p}({\bf{x}}) + \sum\limits_{i = 1}^2 {{{{\gamma _i}} \over {{\eta _i}}}}  + \sum\limits_{i = 1}^{n + 1} {{{{\beta _i}} \over {{W_i}}}} } \right)  \cr 
  & \quad  + H({\bf{x}})\left( {{\delta _p}{{\dot{V}}_p}({\bf{x}}) - \sum\limits_{i = 1}^2 {{{{\gamma _i}} \over {{\eta _i}^2}}} \mathop {{\eta _i}}\limits^.  - \sum\limits_{i = 1}^{n + 1} {{{{\beta _i}} \over {{W_i}^2}}} \mathop {{W_i}}\limits^. } \right), \cr} $$

which can be simplified as

${\dot L_p}({\bf q}) = \sum\limits_{i = 1}^n {\left( {{f_{{i_p}}}{w_i} + {g_p}v} \right)} ,$

where

(11) $$\matrix{ {f_{{i_p}}}({\bf q}) = \left[ {(y - b)\left( {x - \sum\limits_{k = 1}^{i - 1} {{l_k}} cos\left( {\sum\limits_{j = 1}^k {{\theta _j}} } \right)} \right) - (x - a)\left( {y - \sum\limits_{k = 1}^{i - 1} {{l_k}} sin\left( {\sum\limits_{j = 1}^k {{\theta _j}} } \right)} \right)} \right] \cr \left( {\alpha + {\delta _p}{V_p}({\bf x}) + \sum\limits_{k = 1}^2 {\displaystyle{{{\gamma _k}} \over {{\eta _k}}}} + \sum\limits_{k = 1}^{n + 1} {\displaystyle{{{\beta _k}} \over {{W_k}}}} } \right) + H({\bf x})\left( {\sum _{\matrix{ {k = i,} \cr {i \lt 2} \cr } }^2\displaystyle{{{\beta _k}} \over {W_k^2}} + \sum _{\matrix{ {k = i,} \cr {i \gt 1} \cr } }^i\displaystyle{{{\beta _{k + 1}}} \over {W_{k + 1}^2}}} \right) + {\delta _p}H({\bf x}) \cr \left[ {(y - {y_{L{M_p}}})\left( {x - \sum\limits_{k = 1}^{i - 1} {{l_k}} cos\left( {\sum\limits_{j = 1}^k {{\theta _j}} } \right)} \right) - (x - {x_{L{M_p}}})\left( {y - \sum\limits_{k = 1}^{i - 1} {{l_k}} sin\left( {\sum\limits_{j = 1}^k {{\theta _j}} } \right)} \right)} \right], } $$

and

(12) $$\matrix{ {{g_p}({\bf q})}& { = \left( {\alpha + {\delta _p}{V_p}({\bf x}) + \sum\limits_{k = 1}^2 {\displaystyle{{{\gamma _k}} \over {{\eta _k}}}} + \sum\limits_{k = 1}^{n + 1} {\displaystyle{{{\beta _k}} \over {{W_k}}}} } \right)\left( {(x - a)cos\left( {\sum\limits_{k = 1}^n {{\theta _k}} } \right) + (y - b)sin\left( {\sum\limits_{k = 1}^n {{\theta _k}} } \right)} \right)} \cr {} & { + H({\bf x})\sum\limits_{k = 1}^2 {{{( - 1)}^k}} \displaystyle{{{\delta _k}} \over {{\eta _k}}} + {\delta _p}H({\bf x})\left[ {(y - {y_{L{M_p}}})sin\left( {\sum\limits_{k = 1}^n {{\theta _k}} } \right) + (x - {x_{L{M_p}}})cos\left( {\sum\limits_{k = 1}^n {{\theta _k}} } \right)} \right].} \cr {} & {} \cr }$$

Let there be scalars ${\mu _i} > 0$ and $\varphi > 0$. Then, the velocity controllers of system (3) are

(13) $${w_i} = - {\mu _i}{f_{{i_p}}}\;{\rm and}\;v = - \varphi {g_p}.$$

Given (13), system (3) becomes therefore a switched system given as:

(14) $$\mathop {\bf q}\limits^. = {{\bf F}_p}({\bf q}),\;\;{{\bf q}_0}: = {\bf q}({t_0}),\;\;p \in \{ 1,2, \cdots ,m + 1\} .$$

### Stability analysis

The singularities and constraints of the $n$-link revolute manipulator with a prismatic end-effector are converted into artificial obstacles, which are then treated by the Lyapunov based Control Scheme. Therefore, these appear in the controllers as well as the Lyapunov function, ${L_p}({\bf q})$, which is then utilized for the system’s stability analysis. It is clear that ${L_p}({\bf q})$, for $p = \{ 1,2, \cdots ,m + 1\}$, is positive over the domain

$\matrix{ {D({L_p}({\bf q}))}  & {: = }  & {\{ {\bf q} \in {{\mathbb R}^{n + 3}}:{W_i} \gt 0,{\eta _k} \gt 0,\;\forall i = \{ 1,2,3, \ldots ,2(n - 1)\} \;{\rm and}\;k = \{ 1,2\} \} .}  \cr }$

With respect to system (14),

$\matrix{ {{{\dot L}_p}({\bf q})} = { - \sum\limits_{i = 1}^n {\left( {{\mu _i}{f_{{i_p}}}^2 + \varphi {g_p}^2} \right)} \le 0,}  \cr }$

$\forall {\bf q} \in D({L_p}({\bf q}))$. The instantaneous velocities, ${w_i}$ and $v$, are zero at the target, where $(x,y) = (a,b)$ since ${f_{{i_p}}} = 0$ and ${g_p} = 0$. Thus, the end-effector of the *n*-link arm is at the target position. At the target position, the final angular positions of the $n$-links with horizontal axis, and the final length of the prismatic arm are therefore components of an equilibrium point ${{\bf q}_e}$ of system (14). It is explicitly clear that ${L_p}({{\bf q}_e}) = 0$, ${L_p}({\bf q}) > 0\; \forall \; {\bf q} \ne {{\bf q}_e}$ and ${\dot L_p}({\bf q}) \le 0$.

From $S = ({x_0},{y_0}):({p_0},{t_0}),({p_1},{t_1})$ for $p = 1, \ldots ,m + 1$, which is the simple switching sequence of System (14) from which the trajectory is obtained:

$\matrix{ {{{\bf q}_S}( \cdot ): = \{ ({p_0},{t_0}):\mathop {\bf q}\limits^. } = {{{\bf F}_{{p_0}}}({\bf q}(t),t),\;\;p = 1, \ldots ,m + 1,\;{t_0} \le t \lt {t_1}\} .}  \cr }$

Thus, on ${\rm {\cal I}}(S|p)$, ${L_p}({\bf q})$ are monotonically non-increasing. Therefore, ${L_p}$ are Lyapunov-like for ${{\bf F}_p}$ and ${{\bf q}_S}( \cdot )$ over *S*|*p* for $S$ and for all $p$. System (14) is stable in the sense of Lyapunov as per Branicky’s Theorem 2.3 (Branicky, 1998).

${W_k}$, for all $k \in \{ 1,2,3,...,2(n - 1)\}$ and ${\eta _j}$, for $j \in \{ 1,2\}$, are the functions that appear in the denominators of Eqs. (11) and (12). Thus, ${{\bf F}_p}({\bf q}) \in {C^1}[D({L_p}({\bf q})),{{\mathbb R}^2}]$ for all $p = \{ 1,2, \cdots ,m + 1\}$, which signifies that at least on some time interval $[{t_0},s]$, $s > 0$, the solution of ${\bf q}(t)$ of system (14) exists and is in $D({L_p}({\bf q}))$. The functions ${W_k}$ and ${\eta _j}$ will appear in the denominators of higher-order partial derivatives, with each derivative continuous on $D({L_p}({\bf q}))$ since the functions appear in the denominators of (11) and (12). This implies that ${{\bf F}_p}({\bf q}) = ( - {\mu _i}{f_{{i_p}}}({\bf q}), - \varphi {g_p}({\bf q})) \in {C^\infty }[D({L_p}({\bf q})),{{\mathbb R}^2}]$, indicating the existence of the solution ${\bf q}(t)$ of system (14) on $[{t_0},s + \rho ]$, $\rho > 0$ is independent of $s > 0$. Hence, it can be concluded that ${{\bf F}_p}({\bf q})$ is globally Lipschitz continuous on $D({L_p}({\bf q}))$. As a result, system (14) is stable for an $n \in {\mathbb N}$ link revolute arm with a prismatic end-effector for hierarchal landmark navigation.

## Simulation Results

Using Wolfram Mathematica 11.2 software, the computer simulations were generated to validate the results. A number of sequential Mathematica commands were executed to achieve the simulation results. Before the algorithm is executed, the values of the convergence, system singularities and restriction avoidance parameters have to be stated using the brute-force technique. Based on the target position, there can only be an invariant set of initial conditions to facilitate a smooth trajectory of the end-effector as it tracks through the hierarchal landmarks and finally converges to the ultimate target. The limitations and singularities of the mechanical system and the velocity controllers enable the robotic arm to track the desired path. While certain paths are not possible, the set of initial conditions and singularities will change and depend on the path which lead to the target through the LbCS’s notion of steepest descend. Table 1 shows the parameters that need to be defined.

**Table 1:** Table of parameters.

Number of landmarks	$m \in {\mathbb N}$
Position of the ${p^{th}}$ landmarks	$({x_{L{M_p}}},{y_{L{M_p}}}),p \in \{ 1,2, \ldots ,m + 1\}$
Landmark attraction parameters	${\delta _p}$
Number of revolute links	$n \in {\mathbb N}$
Lengths of revolute links	${l_i},i \in \{ 1,2, \ldots ,n\}$
Length of prismatic arm	${r_{max}}$
Initial extension of the prismatic arm	${r_0}$
Prismatic arm restriction parameters	${\gamma _i},\;i \in 1,2$
Initial orientations of revolute links	${\theta _{{i_0}}},\;i \in \{ 1,2, \ldots ,n\}$
Target	$(a,b)$
Target attraction parameter	$\alpha$
Orientation angle restriction parameters of the revolute links	${\beta _i},\;i \in \{ 1,2, \ldots ,n + 1\}$
Revolute link orientation angle convergence parameters	${\mu _i},\;i \in \{ 1,2, \ldots ,n\}$
Prismatic end-effector length convergence parameter	$\varphi$
Maximum possible revolute link orientation angle	${\theta _{{i_{max}}}},\;i \in \{ 2,3, \ldots ,n\}$
Revolute link $1$ limitation angle	$\phi$

The number and positions of the hierarchal landmarks, and initial state of the ${R^n}P$ robotic arm have to be defined in the sequence of commands to be executed. The system (14) was numerically simulated using the RK4 method. At $t = 0$, the initial positions $({x_0}(0),{y_0}(0))$, orientations ${\theta _i}(0)$, and the ${r_0}$ were generated.

### Scenario 1

**Figure 6:**
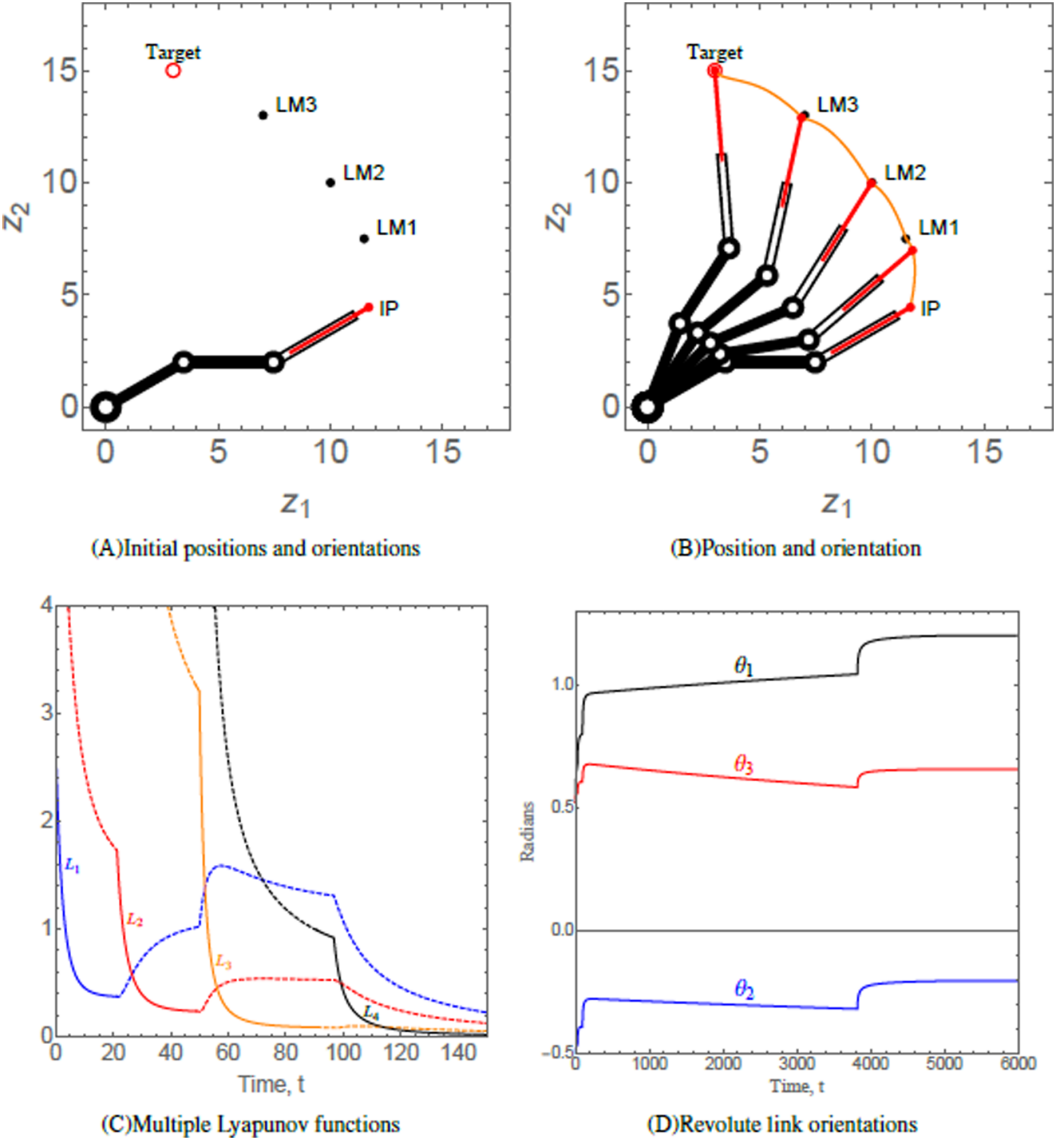
(A) Initial positions and orientations of a 3-link revolute robotic arm with a prismatic end-effector. (B) Positions of the robotic arm at times *t* = 0, 10, 80, 600, and 6,000, respectively. The trajectory of the end-effector is traced in orange. (C) Solid/dashed denotes corresponding system active/inactive. (D) Orientations of the revolute links abiding angular restrictions and limitations.

This scenario considers a 3-link revolute robotic arm with a prismatic end-effector anchored at (0, 0). The robotic arm should maneuver in a manner such that the end-effector navigates through each of the three hierarchal landmarks along its way. The numerical values of the initial states, control parameters, and convergence parameters used are given in Table 2. The initial position (IP) of the end-effector, initial orientation of each of the three revolute links, the positions of three landmarks labelled as LM1, LM2, and LM3 and target of the end-effector of robotic arm are shown in Fig. 6A. As time evolves, the end-effector of robotic arm maneuvers to its target *via* the hierarchal landmarks, as shown in Fig. 6B. This resembles the practical application of robotic arms where a robotic arm should perform various tasks given in hierarchy such as in an assembly line. Figure 6C shows the evolution of the Lyapunov functions ${L_p}$, which is decreasing on each interval on which the ${p^{th}}$ subsystem is active. The behavior of the functions indicate that the end-effector of the robotic arm is converging at each landmark and to its target. The orientation angles of the revolute links are shown in Fig. 6D.

**Table 2:** Numerical values of initial states, control parameters, and convergence parameters.

Position of landmarks	$({x_{L{M_1}}},{y_{L{M_1}}}) = (11.5,7.5)$, $({x_{L{M_2}}},{y_{L{M_2}}}) = (10,10)$, and $({x_{L{M_3}}},{y_{L{M_3}}}) = (7,13)$
Landmark attraction parameters	${\delta _1} = 0.005$, ${\delta _2} = 0.005$, ${\delta _3} = 0.009$, and ${\delta _4} = 0.009$.
Lengths of revolute links	${l_1} = {l_2} = {l_3} = 4$
Length of prismatic arm	${r_{max}} = 4$
Initial extension of the prismatic arm	${r_0} = 0.9$
Prismatic arm restriction parameters	${\gamma _1} = {\gamma _2} = 0.001$
Initial orientations of revolute links	${\theta _{{1_0}}} = {\theta _{{2_0}}} = \displaystyle{\pi \over 6}$ and ${\theta _{{3_0}}} = - \displaystyle{\pi \over 6}$
Target	$(a,b) = (3,15)$
Target attraction parameter	$\alpha = 5 \times {10^{ - 9}}$
Orientation angle restriction parameters of the revolute links	${\beta _1} = {\beta _2} = {\beta _3} = {\beta _4} = 0.001$
Revolute link orientation angle convergence parameters	${\mu _i} = 0.001$ for $i \in \{ 1,2, \ldots ,4\}$
Prismatic end-effector length convergence parameter	$\varphi = 0.6$
Maximum possible revolute link orientation angle	${\theta _{{i_{max}}}} = \displaystyle{{5\pi } \over 6},\;i \in \{ 2,3, \ldots ,n\}$
Revolute link $1$ limitation angle	$\phi = \displaystyle{\pi \over 7}$

### Scenario 2

**Figure 7:**
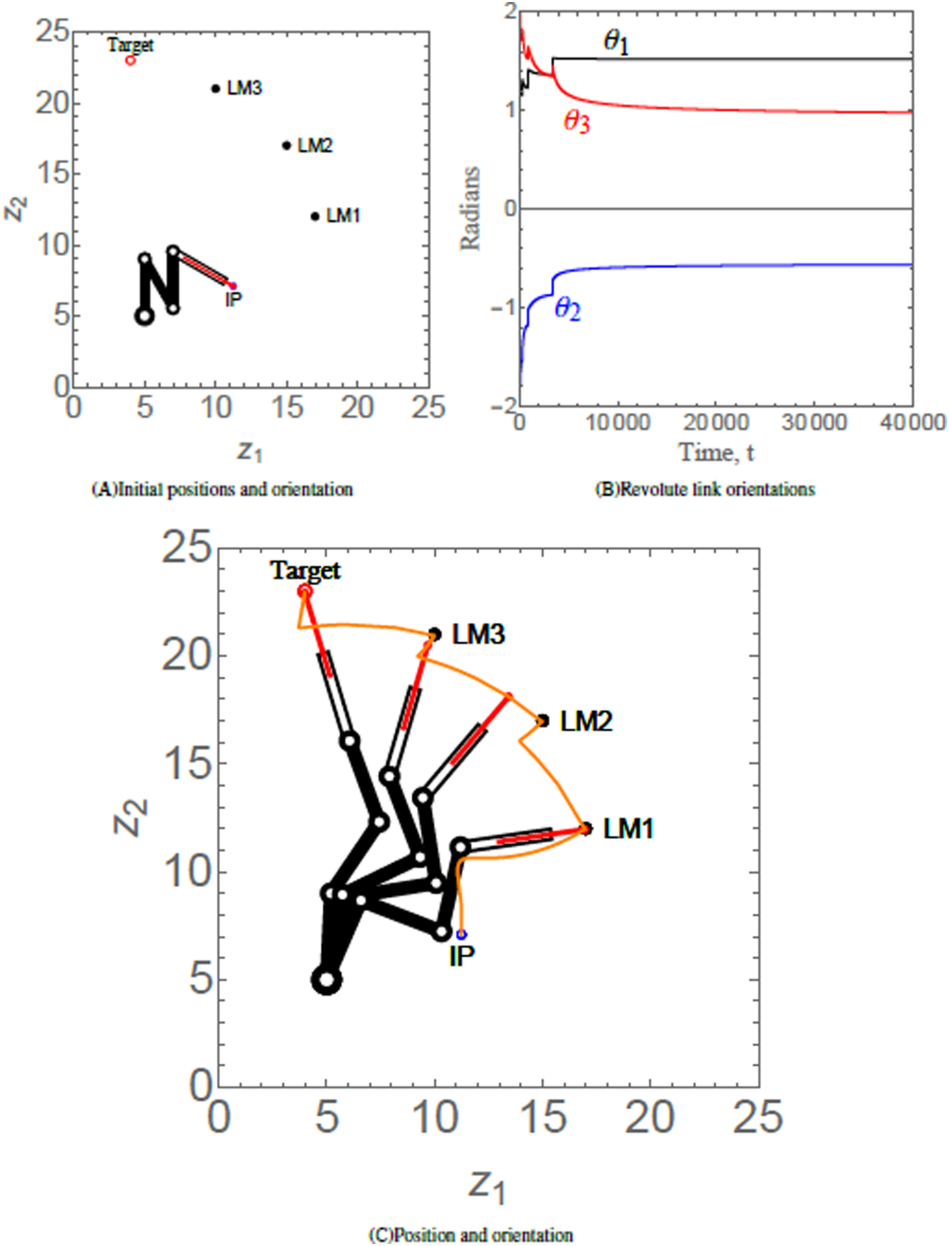
(A) Initial position and orientation of a 4-link revolute robotic arm with a prismatic end-effector. (B) Orientations of the revolute links abiding angular restrictions and limitations. (C) Positions of the robotic arm at times *t* = 320, 903, 1,500, and 200,000, respectively. The trajectory of the end-effector is traced in orange.

Scenario 2 considers a 4-link revolute robotic arm with a prismatic end-effector anchored at (5, 5). The end-effector of the robotic arm has to maneuver to its target *via* three hierarchal landmarks. Table 3 provides the numerical values of the initial states, control, and convergence parameters used for the scenario. Fig. 7A shows the initial position (IP) of the end-effector, initial orientation of each of the four revolute links, the positions of three landmarks labelled as LM1, LM2, and LM3 and target of the end-effector of robotic arm. As time evolves, the end-effector of robotic arm maneuvers to its target *via* the hierarchal landmarks, as shown in Fig, 7B. The evolution of the multiple Lyapunov functions ${L_p}$, which is decreasing on each interval on which the ${p^{th}}$ subsystem is active, is similar to Fig. 6C. The orientations of the revolute links abiding angular restrictions and limitations are shown in Fig. 7C.

**Table 3:** Numerical values of initial states, control parameters, and convergence parameters.

Position of landmarks	$({x_{L{M_1}}},{y_{L{M_1}}}) = (17,12)$, $({x_{L{M_2}}},{y_{L{M_2}}}) = (15,17)$, and $({x_{L{M_3}}},{y_{L{M_3}}}) = (10,21)$
Landmark attraction parameters	${\delta _1} = {\delta _2} = {\delta _3} = 0.1$, and ${\delta _4} = 0.9$.
Lengths of revolute links	${l_1} = {l_2} = {l_3} = {l_4} = 4$
Length of prismatic arm	${r_{max}} = 4$
Initial extension of the prismatic arm	${r_0} = 0.9$
Prismatic arm restriction parameters	${\gamma _1} = {\gamma _2} = 0.001$
Initial orientations of revolute links	${\theta _{{1_0}}} = \displaystyle{\pi \over 2}$, ${\theta _{{2_0}}} = - \displaystyle{\pi \over 6}$, ${\theta _{{3_0}}} = \displaystyle{{5\pi } \over 6}$ and ${\theta _{{4_0}}} = - \displaystyle{{4\pi } \over 6}$
Target	$(a,b) = (4,23)$
Target attraction parameter	$\alpha = 5 \times {10^{ - 14}}$
Parameters for restriction on orientation of the angles revolute links	${\beta _i} = 0.0001$ for $i \in \{ 1,2, \ldots ,6\}$
Revolute link orientation angle convergence parameters	${\mu _i} = 0.00001$ for $i \in \{ 1,2, \ldots ,4\}$
Prismatic end-effector length convergence parameter	$\varphi = 0.0002$
Maximum possible revolute link orientation angle	${\theta _{{i_{max}}}} = \displaystyle{{8\pi } \over 9},\;i \in \{ 2,3,4\}$
Revolute link $1$ limitation angle	$\phi = \displaystyle{\pi \over 7}$

### Scenario 3

**Figure 8:**
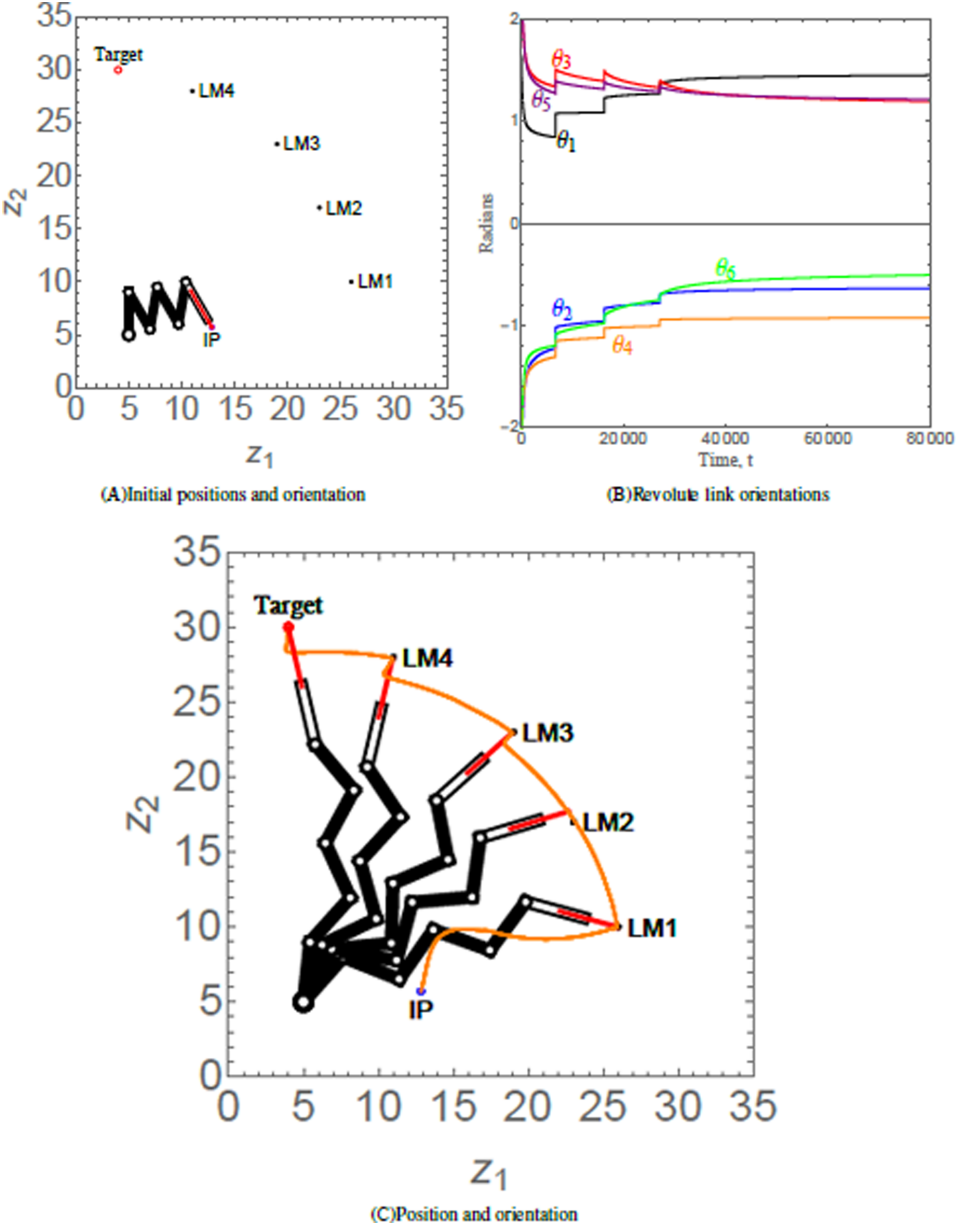
(A) Initial position and orientation of a 6-link revolute robotic arm with a prismatic end-effector. (B) Orientations of the revolute links abiding angular restrictions and limitations. Since the robotic arm has reached its equilibrium state, the orientation angles do not change after *t* = 80,000; hence the revolute link orientations has been curtailed to *t* = 80,000. (C) Positions of the robotic arm at times *t* = 6,695, 6,705, 10,000, 20,000, and 150,000, respectively. The trajectory of the end-effector is traced in orange.

Scenario 3 considers a 6-link revolute robotic arm with a prismatic end-effector anchored at (5, 5). The end-effector of the robotic arm has to maneuver to its target *via* four hierarchal landmarks. Table 4 provides the numerical values of the initial states, control parameters, and convergence parameters used for the scenario. Figure 8A shows the initial position (IP) of the end-effector, initial orientation of each of the six revolute links, the positions of four landmarks labelled as LM1, LM2, LM3, and LM4 and target of the end-effector of robotic arm. As time evolves, the end-effector of robotic arm maneuvers to its target *via* the hierarchal landmarks, as shown in Fig. 8B. The orientation angles of the revolute links are shown in Fig. 8C.

**Table 4:** Numerical values of initial states, control parameters, and convergence parameters.

Position of landmarks	$({x_{L{M_1}}},{y_{L{M_1}}}) = (26,10)$, $({x_{L{M_2}}},{y_{L{M_2}}}) = (23,17)$, $({x_{L{M_3}}},{y_{L{M_3}}}) = (19,23)$ and $({x_{L{M_4}}},{y_{L{M_4}}}) = (11,28)$
Landmark attraction parameters	${\delta _1} = {\delta _2} = {\delta _3} = 0.05$, ${\delta _4} = 0.2$ and ${\delta _5} = 0.8$.
Lengths of revolute links	${l_1} = {l_2} = {l_3} = {l_4} = {l_5} = {l_6} = 4$
Length of prismatic arm	${r_{max}} = 4$
Initial extension of the prismatic arm	${r_0} = 0.9$
Prismatic arm restriction parameters	${\gamma _1} = {\gamma _2} = 0.01$
Initial orientations of revolute links	${\theta _{{1_0}}} = \displaystyle{\pi \over 2},{\theta _{{2_0}}} = - \displaystyle{{5\pi } \over 6},{\theta _{{3_0}}} = {\theta _{{5_0}}} = \displaystyle{{7\pi } \over 9}$ and ${\theta _{{4_0}}} = {\theta _{{6_0}}} = - \displaystyle{{7\pi } \over 9}$
Target	$(a,b) = (4,30)$
Target attraction parameter	$\alpha = 1 \times {10^{ - 14}}$
Parameters for restriction on orientation angles of the revolute links	${\beta _i} = 0.0001$ for $i \in \{ 1,2, \ldots ,7\}$
Revolute link orientation angle convergence parameters	${\mu _i} = 0.00001$ for $i \in \{ 1,2, \ldots ,6\}$
Prismatic end-effector length convergence parameter	$\varphi = 0.001$
Maximum possible revolute link orientation angle	${\theta _{{i_{max}}}} = \displaystyle{{8\pi } \over 9},\;i \in \{ 2,3, \ldots ,6\}$
Revolute link $1$ limitation angle	$\phi = \displaystyle{\pi \over 7}$

**Remark:**

The robotic arms reported in Chen et al. (2019) and Kim, Choi & Kim (2013) can only perform single tasks within a workspace. The proposed system is a modification of previous and existing robotic arms due to its ability to maneuver through multiple hierarchal landmarks, thus accomplishing a sequence of tasks. In addition, the proposed generalized *n*-link robotic arm allows the user to choose, depending on the user’s intended application, a definite number of links (*n*) while using the same control laws. The generalized version is an improvement to the robotic arms reported in the literature, such as Meyer (1993), Sharma, Vanualailai & Prasad (2011), Martinetz, Ritter & Schulten (1990), Iqbal, Islam & Khan (2012) and Asadi et al. (2019), where a specific number of links were utilized. Moreover, mostly revolute links were used in the previous robotic arm systems. The robotic arm proposed in this research is a modified version, with an additional prismatic link.

The simulation results of Scenario 1, Scenario 2 and Scenario 3 portray the robustness of the proposed generalized version of the robotic arm, comprising of *n*-links. As demonstrated, the robotic arms have to maneuver to their targets, proceeding through each of the hierarchal landmarks along the way. Under the proposed controllers, it was observed that the robotic arm successfully reached its target, having a safe and smooth trajectory in all three scenarios. Furthermore, the behavior of the Lyapunov functions indicate that the robotic arms had no difficulty in converging to their targets.

## Discussion

The introduction of robotic arms has eased repetitive assembly line works in our industry sector. The advantages of robotic arm systems in repetitive tasks are that: tasks could be accurately and constantly repeated with a fast speed without getting the robotic arm fatigued, robotic arms can operate under immense conditions, and are cost effective. In this paper, a set of nonlinear, time-invariant, and switched stabilizing velocity controllers of an anchored *n*-link revolute robotic arm has been established to navigate *via* hierarchal landmarks while observing system restrictions and limitations. The controllers allow the robotic arm to perform a sequence of tasks with one complete movement of the articulated arm. Simulation results such as the ones shown in Figs. 6–8 show the controllers’ effectiveness and system robustness for navigation observing system restrictions and limitations.

The LbCS method guarantees stable and controlled motion. In comparison, the inverse kinematics method utilized in Jack et al. (1993) is complex because of the nonlinearities, implying multiple or infinite solutions may exist, and a closed-form solution may not be found. Even if the inverse kinematics has a closed-form solution, unstable movements may happen near the singularities. A significant difficulty in solving inverse kinematics is associated with costly derivation and programming of the algorithms. Moreover, the size and structure of neural networks, as utilized in Guez & Ahmad (1988), also makes it computationally expensive. To reduce the computational complexity of inverse kinematics and neural networks approach, the LbCS method is used in this research. Designing controllers with LbCS, which falls under the artificial potential field method, is easy, and the controllers are continuous. One of the main advantages of LbCS is the treatment of singularities and dynamic constraints through artificial obstacles (Sharma, Vanualailai & Singh, 2015; Sharma et al., 2018; Kumar et al., 2021; Sharma, Raj & Vanualailai, 2018). In previous works on artificial potential fields for robot navigation, a single attraction point has been used (Cosío & Castaneda, 2004). In this study, LbCS has been utilized to guide the robotic arm to track multiple attraction points in the form of hierarchal landmarks before finally converging to the ultimate target. Moreover, the Lyapunov stability theory could be applied with other motion planning techniques such as the closed-loop output feedback control method proposed in Chen & Sun (2021) to obtain asymptotic stability for such mechanical systems assigned to perform multiple tasks *via* hierarchal landmarks.

This has provided a solution to the common problem tagged to robotic arms that require certain tasks that need to be addressed in an hierarchal order. For instance, robotic arms in automated assembly line could perform a number of tasks which are in hierarchal order.

## Conclusion

Stabilizing two-dimensional switched velocity-based controllers were proposed for an *n*-link revolute robotic arm with a prismatic end-effector under hierarchal landmark navigation. The nonlinear time-invariant switched controllers enabled the robotic arm end-effector, governed by its kinematic equations, to navigate from its initial configuration to perform multiple tasks in one sequence *via* hierarchal landmarks while observing the system restrictions and limitations. From the authors’ point of view, this is the first time such stabilizing switched velocity-based controllers are derived for an *n*-link revolute robotic arm with a prismatic end-effector in the sense of Lyapunov.

This paper is a theoretical exposition into the applicability of LbCS, and we have restricted ourselves to showing the effectiveness of velocity-based control laws numerically, using computer-based simulations of interesting scenarios. The drawback of this approach is that algorithm singularities (local minima) can be introduced. In practical applications, continuity has to be discretized, and only asymptotic stability could be shown. It is feasible for the industry sector to include such controllers for the development of autonomous robotic arms which could perform a sequence of tasks provided in a hierarchal order within a single movement. For the future research, control laws for synchronous and asynchronous revolute manipulator with prismatic end-effector for landmark navigation will be considered with experimental prototype robots.

## Supplemental Information

10.7717/peerj-cs.885/supp-1 Supplemental Information 1 Mathematica codes verifying the switched controllers.

## Additional Information and Declarations

### Data Availability

The following information was supplied regarding data availability: The Mathematica codes, which were done in Wolfram Mathematica 12.1 software for verification of the switched controllers of *n*-link revolute robotic arm with prismatic end effector for landmark navigation, are available in the Supplemental File.
